# Is There a Relation between 677C>T Polymorphism in the *MTHFR* Gene and the Susceptibility to Epilepsy in Young Patients? A Meta-Analysis

**DOI:** 10.3390/brainsci11101327

**Published:** 2021-10-06

**Authors:** Beata Sarecka-Hujar

**Affiliations:** Department of Basic Biomedical Science, Faculty of Pharmaceutical Sciences in Sosnowiec, Medical University of Silesia in Katowice, 41-200 Sosnowiec, Poland; bsarecka-hujar@sum.edu.pl; Tel.: +48-32-2699-830

**Keywords:** polymorphism, *MTHFR*, epilepsy, adolescents, children, meta-analysis

## Abstract

*Background*: Numerous data show a role for genetic polymorphisms in the development of epilepsy. Previously, the TT genotype of the *MTHFR* 677C>T polymorphism was found to be associated with a decreased leucocyte DNA methylation status. Polymorphisms in the *MTHFR* gene could modify the pharmacodynamics of many drugs. This meta-analysis aimed to assess the relationship between *MTHFR* 677C>T polymorphism and susceptibility to epilepsy in young patients. *Methods*: Available databases (PubMed, Embase, Google Scholar, SciELO, and Medline) were searched using specific keywords. Eight studies, published between 1999 and 2019, with 1678 young patients with epilepsy and 1784 controls, met the inclusion criteria. Apart from the total groups, additional analyses in age subgroups (i.e., young adults and children) were conducted. Statistical analyses were conducted using the RevMan 5.4 and MedCalc software. The pooled odds ratio (OR) was estimated with a random- or fixed-effects model depending on the heterogeneity. Analyses were performed for five genetic models, i.e., dominant (CT + TT vs. CC), recessive (TT vs. CC + CT), additive (TT vs. CC), heterozygous (CT vs. CC), and allelic (T vs. C). The publication bias was assessed with the use of Egger’s and Begg’s tests. *Results*: Both the *MTHFR* TT genotype (in the additive model) and the T allele (in the allelic model) significantly increased the risk of epilepsy when the total groups were compared (OR = 1.44, *p* = 0.002, and OR = 1.183, *p* = 0.001, respectively). The sensitivity analysis for these models indicated the stability of the results. Similarly, significant results were obtained among young adults for all the genetic models (dominant model: OR = 1.28, *p* = 0.002; recessive model: OR = 1.48, *p* = 0.003; additive model: OR = 1.63, *p* < 0.001; heterozygous model: OR = 1.21, *p* = 0.028; and allelic model: OR = 1.256, *p* < 0.001). Those results were also stable and reliable. In the group of children, no relation between 677C>T polymorphism and epilepsy was observed; however, the analysis was based only on three studies, and one study also comprised young adults. No publication bias was demonstrated. *Conclusions*: The meta-analysis revealed that the carrier state for the T allele as well as the TT genotype of the *MTHFR* 677C>T polymorphism increases the risk of epilepsy in young adults but not in children.

## 1. Introduction

The pathogenesis of epilepsy, which is one of the most common neurological disorders worldwide, is heterogeneous but still not fully known. Epileptogenesis is a dynamic process that begins with the action of one or several damaging factors (e.g., metabolic factors, genetic defects or trauma). In response to the damage factor within the latent period, the changes begin at the molecular level in gene expression and protein synthesis, and then, disturbances in the functioning of ion channels and neurons begin. The consequence of these changes is epileptic seizure as a clinical manifestation [1]. Based on a clinical phenomenology of the seizures, as well as electroencephalography (EEG) and neuroimaging recordings, seizures can be classified as focal, generalized and those of unknown onset. According to their first feature, motor or non-motor seizures can be distinguished. Furthermore, focal seizures may be classified into focal aware seizures and focal impaired-awareness seizures. In turn, epilepsy may be of focal, generalized, combined focal and generalized, and unknown type. Focal and generalized epilepsies are diagnosed based on clinical background, while, when information on the epilepsy type is lacking, the term “unknown” can be used [2].

The first remarks on the hereditary component in epilepsy were emphasized in 400 years BC by Hippocrates, who was also the first one who suggested a possible etiology and therapy for the disease [3]. Due to the development of genetic techniques over the last few decades, the impact of genetic background in epilepsy has been explored more extensively. The study by Vadlamudi et al. [4] based on 384 twin pairs confirmed genetic influences for specific epilepsies. Almost 11% of the analyzed twin pairs had mutations of large effect in known epilepsy genes or carried validated susceptibility alleles [4]. The latest data also demonstrate that genetic polymorphisms have an important role in the development of epilepsy [5].

In experimental animal models, the genome-wide alteration of DNA methylation signatures was demonstrated as a general pathomechanism associated with epileptogenesis and epilepsy [6]. The authors observed increased methylation in genes as well as in a large number of differentially methylated non-genic regions [6]. In human epilepsy, DNA methylation may also differentially regulate certain genes [7,8].

Methylenetetrahydrofolate reductase (MTHFR, EC 1.5.1.20) is an enzyme involved in the remethylation process. MTHFR catalyzes the reduction of 5,10-methyltetrahydrofolate to 5-methyltetrahydrofolate, the dominant form of folate and carbon donor in the remethylation of homocysteine to methionine. The gene encoding MTHFR is located on chromosome 1, in the p36.3 region. The common 677C>T polymorphism within the *MTHFR* gene was found to be related to the thermolability of the enzyme, which, in consequence, leads to an elevated level of homocysteine. Previously, it was observed that a disturbance in homocysteine metabolism may influence cellular methylation processes, including DNA methylation [9]. In the study by Castro et al. [9], the *MTHFR* TT genotype was associated with a decreased leucocyte DNA methylation status. Polymorphisms within the *MTHFR* gene could modify the pharmacodynamics of many drugs that require methylation reactions during metabolism or their biochemical effects [10]. Vilaseca et al. [11] observed the lowest folate levels in carbamazepine-treated patients with the *MTHFR* TT genotype. On the other hand, the authors reported no genotype-dependent effect of carbamazepine treatment on the levels of the B6 and B12 vitamins. Additionally, the effect of valproic acid on the levels of vitamins was not related to *MTHFR* genotypes [11]. Some studies have shown a relation between *MTHFR* 677C>T polymorphism and epilepsy, while others have not [5,11,12,13,14]. In addition, the ages of the patients with epilepsy analyzed in these studies ranged from infancy to adulthood.

To overcome the discrepancies of the results published, a meta-analysis was conducted to assess the relationship between 677C>T polymorphism in the *MTHFR* gene and susceptibility to epilepsy. The attention was focused on young patients, who included children and young adults up to 45 years old. In young people, the involvement of genes in the development of the disease may be of greater importance than in older patients.

## 2. Materials and Methods

### 2.1. Search Strategy

**Figure 1:**
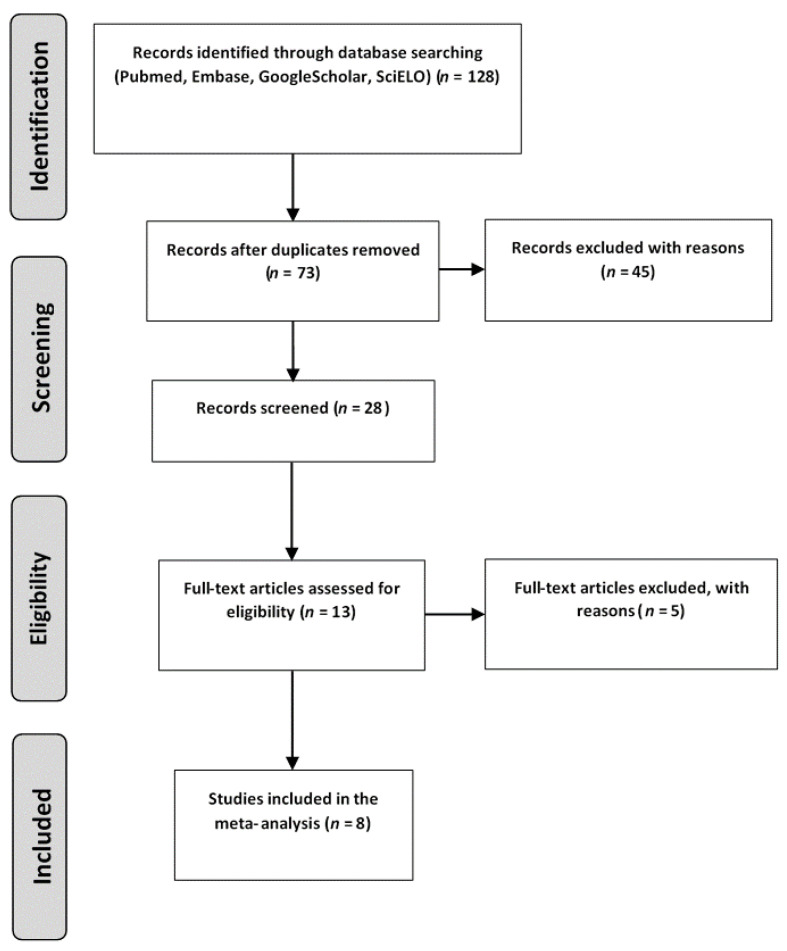
Flow chart presenting the process of searching for the eligible articles.

Databases (PubMed, Embase, Google Scholar, SciELO and Medline) were searched for relevant papers (the last search was performed in late August 2021) using the following keywords: (“*MTHFR* polymorphism” or “677C>T polymorphism”) and (“epilepsy” or “antiepileptic drugs”) and (“young adults” or “adolescents” or “children” or “infants”). The studies searched were included in the meta-analysis when the following criteria were met: (a) confirmed epilepsy, (b) patients and control groups, (c) ages of the patients younger than 45 years old, (d) access to data on genotypes, (e) full-length paper or brief communication, and (f) article written in English. In turn, publications were excluded for the following reasons: (a) unavailability of genotyping results; (b) lack of a reference (control) group; (c) mean ages of the epilepsy patients above 45 years; (d) conference proceedings, review articles, case reports, meta-analyses or animal studies as article types; and (e) language of the article other than English. Eventually, 8 case–control studies [5,11,12,13,14,15,16,17] published from 1999 to 2019, with a total number of 1678 young patients with epilepsy and 1784 controls, met the inclusion criteria. Figure 1 shows a flow diagram for the process regarding the search and reasons for excluding the studies according to the PRISMA guidelines [18].

### 2.2. Data Extraction and Methodological Quality

The following data were extracted from each study included in the present meta-analysis: the first author’s name, year of publication, population, ages of cases and control subjects, and sizes of the analyzed groups, as well as the number with a particular genotype of the *MTHFR* 677C>T polymorphism in both the subjects and controls. If applicable, the number of *MTHFR* alleles was also extracted; otherwise, the alleles were calculated based on genotype frequencies. To establish the methodological quality of the studies, an assessment with the Newcastle–Ottawa scale (NOS) for case–control studies was performed [19]. Using the NOS scale, a study can be scored within the range of 0 to 11 points. When an article achieved at least 5 points, it was considered of sufficient quality. The Hardy–Weinberg equilibrium (HWE) for controls was calculated in each study.

### 2.3. Statistical Analyses

Statistical analyses were conducted twice with the use of the Review Manager software (RevMan version 5.4; Cochrane, London, UK) and MedCalc software (version 19.5.3.; MedCalc Software Ltd., Ostend, Belgium), and an experienced biostatistician served to solve doubts. To determine the strength of association between the selected genetic model and the disease, the pooled odds ratio (OR) with 95% confidence intervals (CIs) was assessed. To assess the degree of heterogeneity between the studies included, the I^2^ was calculated. It describes the proportion of variance (from 0% to 100%) that is due to variance in the true effect sizes rather than sampling error. The selection of the statistical model for the analyses was performed on the basis of heterogeneity. I^2^ at 25%, 50%, and 75% suggested low, intermediate, and high inconsistency, respectively. In the case of significant heterogeneity between studies, the random-effects method (DerSimonian–Laird; REM) was used to calculate the pooled OR with the 95% CI, whereas in the case of nonsignificant heterogeneity, the calculation was performed with the fixed-effects method (Mantel–Haenszel, FEM). The strength of the correlation between the *MTHFR* 677C>T polymorphism and epilepsy was assessed in the following models: dominant (CT + TT vs. CC), recessive (TT vs. CC + CT), additive (TT vs. CC), heterozygote (CT vs. CC), and allelic (T vs. C).

To assess the potential publication bias, both Egger’s regression and Begg’s rank correlation tests were performed. In addition, to evaluate the stability of the results, sensitivity analyses were performed by the sequential exclusion of each study. The result was considered to be statistically significant when the *p* value was below 0.05.

## 3. Results

### 3.1. Characteristics of the Studies Included

The crucial data extracted from the studies included in the present meta-analysis (i.e., the population, year of publication, sample size, age, and relationship assessed) are shown in Table 1.

In general, the studies were performed in different racial populations, whereas Caucasians were the most numerous. In the case of the age of the studied patients and controls, two studies analyzed only children [5,11]. In turn, the study by Ono et al. [16] analyzed patients from 1 year old to 40 years old; thus, this population contained both children and young adults. In the case of this study, the mean age of the patients was lower than that of the controls, while the age range for the patients was wider compared to that for the control group [16]. In the study by Kini et al. [17], the age of the epileptic patients was not provided; however, the study group consisted of pairs: pregnant women with epilepsy and their babies examined after delivery. Therefore, women analyzed by Kini et al. [17] were assumed to be of reproductive age, which allowed the classification of them as young patients. The largest groups of epilepsy patients and controls were recruited by Scher et al. [14], Al-Eitan et al. [5], and Dean et al. [12] as well as Kini et al. [17]. In turn, the lowest number of patients and control subjects was recruited by Vilaseca et al. [11] and Ono et al. [16].

The correlation between 677C>T polymorphism in the *MTHFR* gene was observed in three studies [12,14,15]. In the study by Ono et al. [16], the relationship of *MTHFR* genotypes with symptomatic or cryptogenic epilepsy but not with idiopathic epilepsy was reported. In turn, Aydin et al. [13] and Vilaseca et al. [11] observed no correlation, whereas AL-Eitan et al. [5] established an association only in a generalized tonic-clonic epilepsy subgroup. In one study, no clear relation was described [17].

The distribution of the genotypes and alleles of the *MTHFR* 677C>T polymorphism, together with the results of the HWE in the controls and quality assessment for each study, is shown in Table 2.

One study [11] demonstrated a lack of agreement with the Hardy–Weinberg equilibrium in the prevalence of genotypes in controls, which is probably due to the low number of subjects in the control group (Table 2). However, the assumption of Minelli et al. [20] was adopted in order not to exclude studies that differed from the HWE.

### 3.2. Association between 677C>T Polymorphism in MTHFR Gene and Epilepsy in Total Groups

The TT genotype of the *MTHFR* gene was present in over 12% of the total group of epileptic young patients compared to almost 10% of the healthy controls. Additionally, carriers of the T allele (subjects with CT or TT genotypes) were more frequent in the epilepsy group than in the controls (55% vs. 51%).

**Figure 2:**
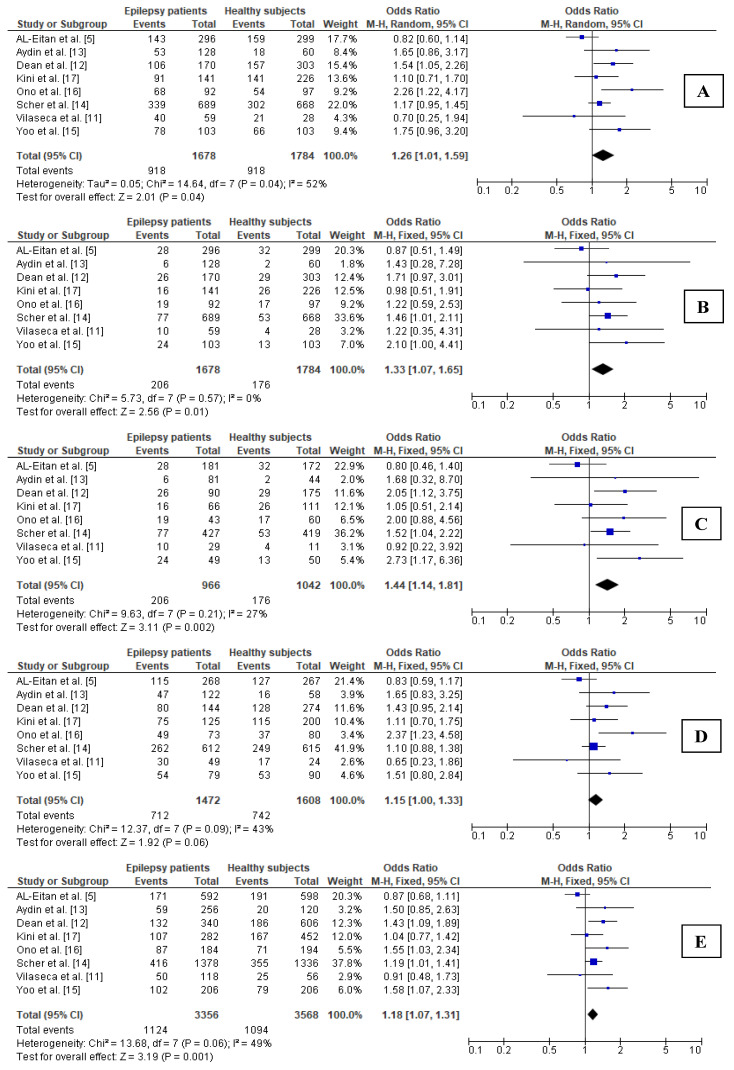
Forest plots for relations between different genetic models of *MTHFR* polymorphism and epilepsy in total groups: (**A**) CT + TT vs. CC; (**B**) TT vs. CT + CC; (**C**) TT vs. CC; (**D**) CT vs. CC; (**E**) T vs. C. M.-H.—Mantel–Haenszel; CI—confidence interval; I^2^—heterogeneity; df—degrees of freedom.

Significant heterogeneity between the analyzed studies was observed only in the case of the dominant model (i.e., *MTHFR* CT + TT vs. CC) for the total groups (I^2^ = 52%; *p* = 0.04); thus, the pooled OR was calculated using the REM (Figure 2). In other genetic models, no heterogeneity was demonstrated, which allowed the use of FEM. The results of the statistical analyses showed that the carrier state for the *MTHFR* 677T allele may be related to epilepsy (OR = 1.26; 95% CI 1.01–1.59; *p* = 0.04) (Figure 2). The recessive model (i.e., TT vs. CT + CC) also revealed a significant difference in the prevalence of TT homozygotes in comparison to CC and CT genotypes between the analyzed groups of young patients (OR = 1.33; 95% CI 1.07–1.65; *p* = 0.01).

Similarly, in the additive model (i.e., the comparison of TT homozygotes vs. CC homozygotes), the results were statistically significant (OR = 1.44; 95% CI 1.14–1.81; *p* = 0.002). A weaker relation, but again significant, was observed for the allelic model (T vs. C; OR = 1.18; 95% CI 1.07–1.31; *p* = 0.001), whereas a lack of relation was demonstrated for the heterozygous model (CT vs. CC, *p* = 0.06).

### 3.3. Sensitivity Analysis for Comparisons in Total Groups

In the sensitivity analysis, no change in the OR value was demonstrated in the case of additive, heterozygous, and allelic models after excluding subsequent studies. Therefore, these analyses were considered stable. However, in the dominant model after omitting both the study by Scher et al. [14] and the study by Yoo et al. [16], the significance was lost (OR = 1.22, 95% CI 0.96–1.56, *p* = 0.10, and OR = 1.31, 95% CI 0.96–1.77, *p* = 0.09, respectively, in the REM). In turn, omitting the study by Scher et al. [14] also changed the results in the recessive model of the present meta-analysis into nonsignificant (OR = 1.26; 95% CI 0.96–1.65; *p* = 0.09). Thus, the results in the dominant and recessive model should be treated with caution.

### 3.4. Publication Bias in Total Groups

In the total group, no publication bias was observed since the shapes of the funnel plots of all the genetic models analyzed were roughly symmetrical. Table 3 shows the exact results of both Egger’s and Begg’s tests for all the genetic models in the total groups.

### 3.5. Subgroup Analyses

Due to the wide range of the ages of the epileptic patients from the studies included, two analyses in age subgroups were additionally performed, i.e., in the group of young adults (age over 18 years but below 45 years) and in the group of children.

#### 3.5.1. Association between 677C>T Polymorphism in *MTHFR* Gene and Epilepsy in Young Adults

In this meta-analysis, five studies were included [12,13,14,15,17], with 1231 young patients with epilepsy and 1360 healthy subjects as controls. The proportion of the carriers of the *MTHFR* T allele in the young adults with epilepsy and controls was similar to that observed in the total group (54% vs. 50%, respectively).

**Figure 3:**
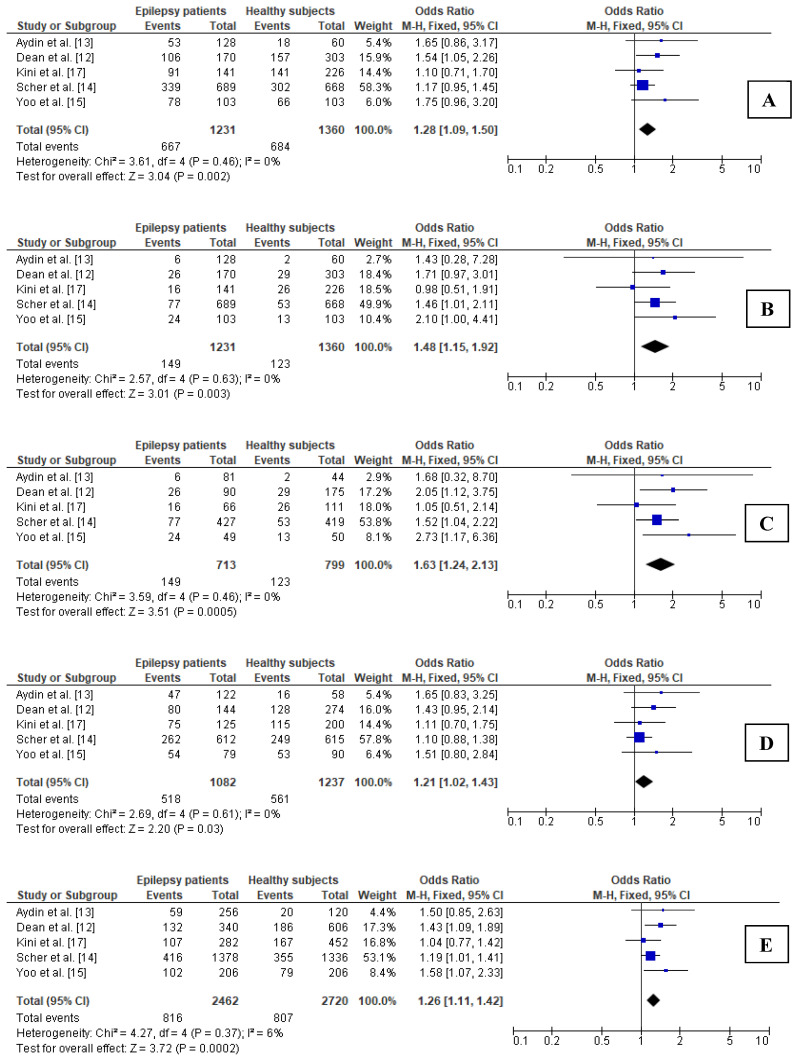
Forest plots for relations between different genetic models of *MTHFR* polymorphism and epilepsy in the subgroup of young adults: (**A**) CT + TT vs. CC; (**B**) TT vs. CT + CC; (**C**) TT vs. CC; (**D**) CT vs. CC; (**E**) T vs. C. M.-H.—Mantel–Haenszel; CI—confidence interval; I^2^—heterogeneity; df—degrees of freedom.

In all the analyzed genetic models for young adults, nonsignificant heterogeneity between the analyzed studies was observed; thus, FEM was used to calculate the OR (Figure 3).

In young adults, the carrier state for the *MTHFR* 677T allele may be a risk factor for epilepsy (OR = 1.28; 95% CI 1.09–1.50; *p* = 0.002). A higher OR was found in the case of the recessive model in young adults (OR = 1.48; 95% CI 1.15–1.92; *p* = 0.003), while the highest OR was shown for the additive model (OR = 1.63; 95% CI 1.24–2.13; *p* < 0.001). In turn, comparable values of pooled OR were demonstrated for both heterozygous as well as allelic models in young adults (OR = 1.21; 95% CI 1.02–1.43; *p* = 0.028, and OR = 1.26; 95% CI 1.11–1.42; *p* < 0.001, respectively).

#### 3.5.2. Sensitivity Analysis for Comparisons in Subgroup of Young Adults

The sequential exclusion of each study included in the meta-analysis in the subgroup of young adults revealed no change in the OR value in the case of any of the genetic models. Thus, the results are stable and reliable.

#### 3.5.3. Publication Bias in Subgroup of Young Adults

Similarly to in the total groups, in the subgroup of young adults, no publication bias was demonstrated (Table 4).

#### 3.5.4. Association between 677C>T Polymorphism in *MTHFR* Gene and Epilepsy in Children

This meta-analysis contained three studies [5,11,16] with an epilepsy group involving 447 children and control group comprising 424 healthy subjects. In this analysis, the frequency of *MTHFR* T allele carriers in children with epilepsy was comparable to that observed in the controls (56% vs. 55%, respectively).

**Figure 4:**
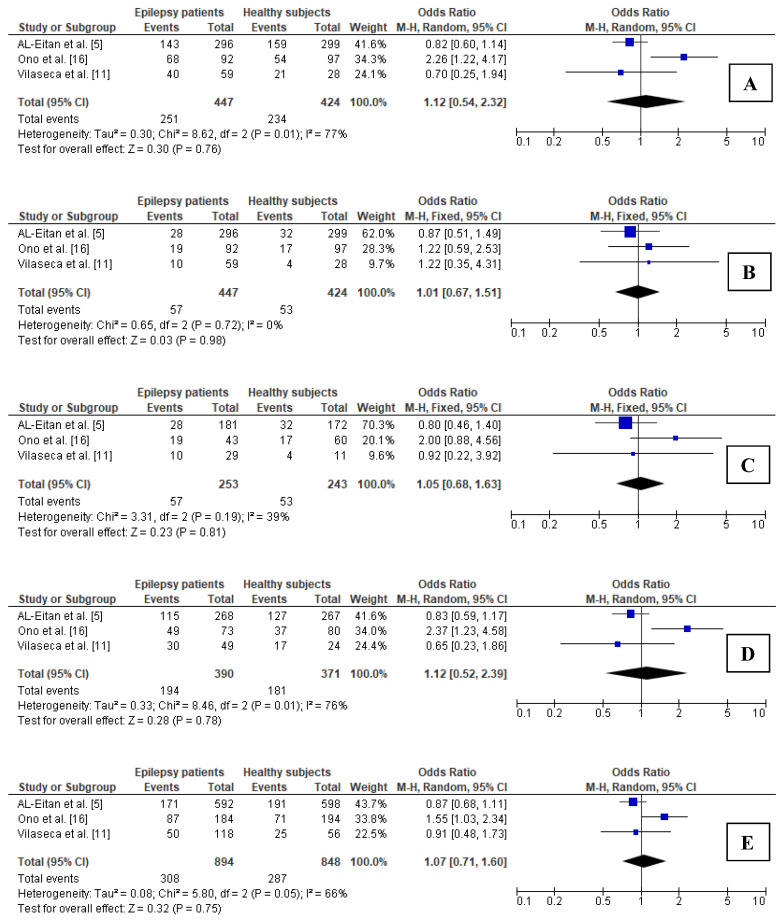
Forest plots for relations between different genetic models of *MTHFR* polymorphism and epilepsy in the subgroup of children: (**A**) CT + TT vs. CC; (**B**) TT vs. CT + CC; (**C**) TT vs. CC; (**D**) CT vs. CC; (**E**) T vs. C. M.-H.—Mantel–Haenszel; CI—confidence interval; I^2^—heterogeneity; df—degrees of freedom.

Significant heterogeneity between the analyzed studies was observed for the dominant as well as heterozygous model; thus, the random-effects model was used to calculate the pooled OR. In the children subgroup, none of the analyzed genetic models showed significance (Figure 4).

#### 3.5.5. Sensitivity Analysis for Comparisons in Subgroup of Children

The sequential exclusion of each study included in the meta-analysis in the subgroup of children demonstrated that the values of the OR in the case of any of the genetic models did not change. Thus, the results are stable and reliable.

#### 3.5.6. Publication Bias in Subgroup of Children

In the subgroup of children, no publication bias was demonstrated, which was consistent with the results in the total groups and in the subgroup of young adults (Table 5).

## 4. Discussion

The updated meta-analysis revealed that, in the total groups, the *MTHFR* TT genotype may be related to epilepsy in comparison to the CC genotype (i.e., in the additive model, TT vs. CC; OR = 1.44; *p* = 0.002). The sensitivity analysis indicated the results were stable and therefore reliable. Table 6 summarizes the detailed results for all the genetic models in all the groups/subgroups.

The results of the dominant (CT + TT vs. CC) and recessive (TT vs. CT + CC) models were not stable; therefore, despite the fact of significance, they should be treated with caution.

Previously, two meta-analyses analyzing the relation between 677C>T polymorphism in the *MTHFR* gene and epilepsy were published [21,22]. The study by Wu et al. [21] from 2014 included ten studies, while the study by Rai et al. [22] was published in 2018 and included 12 studies, i.e., 10 studies were the same as in Wu et al.’s analysis [21] and two additional studies were included [23,24]. Both of these papers were not included into the present meta-analysis since the study of Balamuralikrishnan et al. [23] was not a full-length paper, just a conference abstract, while the study by Munisamy et al. [24] was conducted on patients with ages ranging from 16 to 60 years old. Due to age, several studies were excluded from the current meta-analysis [25,26,27,28]. Caccamo et al. [25] observed that all patients chronically treated with enzyme-inducing, folate-depleting antiepileptic drugs and simultaneously bearing the 677 TT genotype and 1298 AA genotype or 677 CT and 1298 AA genotypes of the *MTHFR* gene had elevated plasma levels of homocysteine. Earlier data demonstrated that the inhibitory effect of homocysteine thiolactone on Na+/K ATPase activity in the hippocampus cells of male rats can cause seizures [29]. Since the *MTHFR* gene may affect different transcriptomes and proteomes, it may influence the clinical response to various drugs, e.g., antiepileptic drugs [30]. In the study by Ullah et al. [30], epilepsy patients from a Pakhtun population (Pakistan) who were heterozygotes for both common polymorphisms of the *MTHFR* gene had poor seizure control. In the study by Kini et al. [17], no relation between *MTHFR* 677C>T polymorphism and epilepsy itself was established since the authors analyzed the impact of the *MTHFR* genotype on the rate of major malformations in the offspring of women taking antiepileptic drugs as a secondary outcome. The study demonstrated that neither controls carrying the T allele (CT+TT genotypes) nor cases exposed to antiepileptic drugs having the CC genotype had a higher risk of major malformations [17].

After dividing the studies involved in the present paper according to age, greater odds for the correlation between *MTHFR* 677C>T polymorphism and epilepsy among young adults were demonstrated within all the analyzed genetic models, especially for the recessive model (OR = 1.48; 95% CI 1.15–1.92; *p* = 0.003) and additive model (OR = 1.63; 95% CI 1.24–2.13; *p* < 0.001). The results were stable and reliable. The present meta-analysis is the first one to include a children subgroup. However, no relation between 677C>T polymorphism and epilepsy in the children groups was observed. The analysis should be treated with caution since it was based on only three studies, and one study also comprised young adults. The study by Ono et al. [16] was included in the children subgroup due to the fact that the ages of the cases ranged from 1 to 40 years (mean age: 14.8 years), although the authors did not provide information on the proportion of children/young adults. No publication bias was demonstrated for the children subgroup. Two articles based on pediatric patients were excluded from the meta-analysis since no information on the genetic distribution in the controls was found [31,32]. Vurucu et al. [32] demonstrated that 677C>T polymorphism of the *MTHFR* gene had no impact on elevated levels of homocysteine in epileptic children receiving carbamazepine or valproic acid. In addition, a recent study by Zhu et al. [33] analyzed *MTHFR* polymorphism in epileptic patients and controls aged 15–55 years old but in reference to its impact on homocysteine levels. The authors did not demonstrate genotype distributions. However, in the study, the *MTHFR* TT genotype was observed to increase patients’ susceptibility to the effect of oxcarbazepine in disrupting homocysteine homeostasis [33].

The strength of the present study is the analysis within age subgroups, which was performed for the first time. This approach showed that the 677C>T polymorphism may be related to epilepsy in young adults, whereas including children in the overall analysis lowered the results. Therefore, it can be assumed that the analyses regarding the relationship of the 677C> T polymorphism in the *MTHFR* gene should be carried out in strictly defined age groups, so that the results are not underestimated or overestimated.

In the present study, some limitations should be addressed. The first one is the small number of studies included in the meta-analysis. Secondly, the analysis was generally conducted for epilepsy; there was no possibility to perform analyses in specific epilepsy subgroups. In addition, there were also no additional data on other factors that could possibly interact with the *MTHFR* polymorphism in the development of epilepsy. Performing meta-analyses of interactions between particular genes and factors that are simultaneously present in the patients would be more accurate for understanding the role of the polymorphism in the pathogenesis of the disease. In the case of *MTHFR* polymorphism, the level of homocysteine should be especially considered, as the presence of a particular *MTHFR* genotype without elevated levels of homocysteine may have no impact on the pathogenesis of epilepsy.

## 5. Conclusions

Performing research to identify possible genetic biomarkers that will be clinically useful may be of great importance from the point of view of the diagnosis and treatment of epilepsy. The present meta-analysis revealed that the carrier state for the *MTHFR* 677T allele as well as the TT genotype in comparison with both the CC genotype and CC + CT genotypes is related to epilepsy in young adults, but no such correlations were observed for the pediatric population.

## Figures and Tables

**Table 1:** Characteristics of the studies included in the meta-analysis.

Study	Year of Publication	Population	Epilepsy Group *n*	Age (Years)	Control Group*n*	Age (Years)	Established Relationship
AL-Eitan et al. [5]	2019	Jordan	269	Under 15	299	5.9 ± 3.8	No (in the total group) Yes (in generalized epilepsy subgroup)
Vilaseca et al. [11]	2000	Spain	59	Range: 1–18	28	Age-matched to cases	No
Dean et a. [12]	2008	Scotland	170	NA (but both groups were age-matched)	303	29.0 ± 11.3	Yes
Aydin et al. [13]	2017	Turkey	128	27.6 ± 16.0	60	Age-matched to cases	No
Scher et al. [14]	2011	USA	689	32.0 ± 8.5	668	32.0 ± 8.5	Yes
Yoo et al. [15]	1999	South Korea	103	27.5 ± 8.5	103	28.1 ± 9.8	Yes
Ono et al. [16]	2000	Japan	92	Mean: 14.8 (range: 1–40)	97	Mean: 25 (range: 20–30)	Yes (in symptomatic or cryptogenic epilepsy)
Kini et al. [17]	2007	UK	141	NA	226	Age-matched to cases	NA
Total			1678		1784		

M—male; F—female; NA—not applicable.

**Table 2:** Distribution of genotypes and alleles of *MTHFR* 677C>T polymorphism, as well as HWE and quality assessments, in the studies included.

Study	Epilepsy Group		Control Group		HWE (for Controls) (χ^2^; *p*)	NOS
	Genotypes of *MTHFR* 677C>T Polymorphism *n* (%)	Alleles of *MTHFR* 677C>T Polymorphism *n* (%)	Genotypes of *MTHFR* 677C>T Polymorphism *n* (%)	Alleles of *MTHFR* 677C>T Polymorphism *n* (%)		
AL-Eitan et al. [5]	CC: 153 (51.7) CT: 115 (38.8) TT: 28 (9.5)	C: 421 (71.1) T: 171 (28.9)	CC: 140 (46.8) CT: 127 (42.5) TT: 32 (10.7)	C: 407 (68.1) T: 191 (31.9)	0.051; 0.82	8
Vilaseca et al. [11]	CC: 19 (32.2) CT: 30 (50.8) TT: 10 (17.0)	C: 68 (57.6) T: 50 (42.4)	CC: 7 (25.0) CT: 17 (60.7) TT: 4 (14.3)	C: 31 (55.4) T: 25 (44.6)	5.022; 0.02	6
Dean et a. [12]	CC: 64 (37.6) CT: 80 (47.1) TT: 26 (15.3)	C: 208 (61.2) T: 132 (38.8)	CC: 146 (48.2) CT: 128 (42.2) TT: 29 (9.6)	C: 420 (69.3) T: 186 (30.7)	0.007; 0.93	7
Aydin et al. [13]	CC: 75 (58.6) CT: 47 (36.7) TT: 6 (4.7)	C: 197 (76.9) T: 59 (23.1)	CC: 42 (70.0) CT: 16 (26.7) TT: 2 (3.3)	C: 100 (83.3) T: 20 (16.7)	0.145; 0.70	7
Scher et al. [14]	CC: 350 (50.8) CT: 262 (38.0) TT: 77 (11.2)	C: 962 (69.8) T: 416 (30.2)	CC: 366 (54.8) CT: 249 (37.3) TT: 53 (7.9)	C: 981 (73.4) T: 355 (26.6)	0.190; 0.66	7
Yoo et al. [15]	CC: 25 (24.3) CT: 54 (52.4) TT: 24 (23.3)	C: 104 (50.5) T: 102 (49.5)	CC: 37 (35.9) CT: 53 (51.5) TT: 13 (12.6)	C: 127 (61.6) T: 79 (38.4)	0.794; 0.37	6
Ono et al. [16]	CC: 24 (26.1) CT: 49 (53.3) TT: 19 (20.6)	C: 97 (52.7) T: 87 (47.3)	CC: 43 (44.4) CT: 37 (38.1) TT: 17 (17.5)	C: 123 (63.4) T: 71 (36.6)	0.007; 0.93	5
Kini et al. [17]	CC: 50 (35.5) CT: 75 (53.2) TT: 16 (11.3)	C: 175 (62.1) T: 107 (37.9)	CC: 85 (37.6) CT: 115 (50.9) TT: 26 (11.5)	C: 285 (63.1) T: 167 (36.9)	0.854; 0.36	7
Total	CC: 760 (45.3) CT: 712 (42.4) TT: 206 (12.3)	C: 2232 (66.5) T: 1124 (33.5)	CC: 866 (48.5) CT: 742 (41.6) TT: 176 (9.9)	C: 2474 (69.3) T: 1094 (30.7)		

HWE—Hardy–Weinberg equilibrium; NOS—Newcastle–Ottawa Scale.

**Table 3:** The results of Egger’s and Begg’s tests for all genetic models in total groups.

Genetic Model	Egger’s Test			Begg’s Test	
	Intercept	95% CI	*p*	Kendall’s Tau	*p*
Dominant	1.023	−1.956 to 4.002	0.433	0.071	0.805
Recessive	−0.075	−2.304 to 2.153	0.937	0.214	0.458
Additive	0.216	−2.522 to 2.955	0.853	0.143	0.621
Heterozygous	1.069	−1.660 to 3.797	0.375	0.214	0.458
Allelic	0.784	−2.467 to 4.036	0.577	0.071	0.805

CI—confidence interval.

**Table 4:** The results of Egger’s and Begg’s tests for all genetic models in the subgroup of young adults.

Genetic Model	Egger’s Test			Begg’s Test	
	Intercept	95% CI	*p*	Kendall’s Tau	*p*
Dominant	1.485	−1.202 to 4.171	0.177	0.400	0.327
Recessive	0.074	−3.269 to 3.418	0.948	0.200	0.624
Additive	0.475	−3.252 to 4.203	0.712	0.200	0.624
Heterozygous	1.498	−0.440 to 3.436	0.091	0.600	0.142
Allelic	1.256	−2.549 to 5.062	0.371	0.200	0.624

CI—confidence interval.

**Table 5:** The results of Egger’s and Begg’s tests for all genetic models in the subgroup of children.

Genetic Model	Egger’s Test			Begg’s Test	
	Intercept	95% CI	*p*	Kendall’s Tau	*p*
Dominant	1.464	−46.030 to 48.958	0.762	0.333	0.602
Recessive	1.100	−11.880 to 14.081	0.476	0.333	0.602
Additive	1.083	−35.202 to 37.368	0.769	0.333	0.602
Heterozygous	1.324	−46.659 to 49.307	0.785	0.333	0.602
Allelic	1.713	−42.493 to 45.919	0.709	0.333	0.602

CI—confidence interval.

**Table 6:** Summary of heterogeneity; effect model used; the results for pooled ORs, 95% CIs, and *p*; and sensitivity analysis (i.e., stability of the results obtained) in all genetic models within all analyzed groups/subgroups.

Genetic Model	Heterogeneity I^2^ (%), *p*	Effect Model	Pooled OR, 95% CI, *p*	Stability of Results (Yes/No)
Total Groups (8 Studies)				
Dominant (CT + TT vs. CC)	52.19, 0.041	random	1.264, 1.005–1.589, **0.045**	No
Recessive (TT vs. CC + CT)	0.00, 0.572	fixed	1.327, 1.069–1.648, **0.010**	No
Additive (TT vs. CC)	27.35, 0.210	fixed	1.440, 1.144–1.812, **0.002**	Yes
Heterozygous (CT vs. CC)	43.39, 0.089	fixed	1.153, 0.997–1.333, 0.055	Yes
Allelic (T vs. C)	48.84, 0.057	fixed	1.183, 1.067–1.311, **0.001**	Yes
**Young adults** **(5 studies)**				
Dominant (CT + TT vs. CC)	0.00, 0.462	fixed	1.281, 1.092–1.503, **0.002**	Yes
Recessive (TT vs. CC + CT)	0.00, 0.632	fixed	1.483, 1.148–1.918, **0.003**	Yes
Additive (TT vs. CC)	0.00, 0.464	fixed	1.627, 1.240–2.135, **<0.001**	Yes
Heterozygous (CT vs. CC)	0.00, 0.611	fixed	1.209, 1.021–1.431, **0.028**	Yes
Allelic (T vs. C)	6.42, 0.370	fixed	1.256, 1.114–1.417, **<0.001**	Yes
**Children** **(3 studies)**				
Dominant (CT + TT vs. CC)	76.80, 0.013	random	1.119, 0.540–2.318, 0.762	Yes
Recessive (TT vs. CC + CT)	0.00, 0.722	fixed	1.006, 0.670–1.510, 0.978	Yes
Additive (TT vs. CC)	39.50, 0.192	fixed	1.054, 0.682–1.628, 0.814	Yes
Heterozygous (CT vs. CC)	76.36, 0.015	random	1.117, 0.521–2.394, 0.777	Yes
Allelic (T vs. C)	65.53, 0.055	random	1.070, 0.710–1.600, 0.750	Yes

OR—odds ratio; CI—confidence interval; Significant results are in bold.

## Data Availability

The data presented in this study are available on request from the Department of Basic Biomedical Science, Faculty of Pharmaceutical Sciences in Sosnowiec, Medical University of Silesia in Katowice (Poland). The data are not publicly available due to privacy restrictions.
